# Anti-fibrillarin Positive Systemic Sclerosis Presenting With Myositis and Perimyocarditis

**DOI:** 10.7759/cureus.117416

**Published:** 2026-10-04

**Authors:** Niamh Heneghan, Gibran Merali, Kate Akester, Anjali Patel

**Affiliations:** 1 General Medicine, University Hospital Southampton NHS Foundation Trust, Southampton, GBR; 2 General Medicine, Southampton General Hospital, Southampton, GBR

**Keywords:** anti-fibrillarin antibody, connective tissue disease, inflammatory myositis, perimyocarditis, systemic sclerosis overlap

## Abstract

A 45-year-old man with no significant past medical history presented with six months of progressive proximal muscle weakness, inflammatory arthralgia, and functional decline, on a background of two years of progressive depigmented pruritic skin lesions. Examination demonstrated widespread hypopigmented sclerotic skin changes, sclerodactyly, and digital ulceration. Investigations revealed mildly elevated creatine kinase (CK) and cardiac biomarkers, with cardiac magnetic resonance imaging (MRI) demonstrating perimyocarditis and thigh MRI showing symmetrical muscle oedema consistent with inflammatory myopathy.

Skin biopsy demonstrated dermal fibrosis while serology was positive for anti-fibrillarin antibodies. A diagnosis of anti-fibrillarin-positive systemic sclerosis with inflammatory myositis was made. Treatment with low-dose prednisolone and colchicine resulted in clinical improvement, with mycophenolate mofetil (MMF) planned as a steroid-sparing agent. Initiation was delayed following a positive QuantiFERON-TB (QIAGEN GmbH, Hilden, Germany) test requiring management of latent tuberculosis (TB). This case highlights the diagnostic complexity of anti-fibrillarin-positive systemic sclerosis presenting with simultaneous muscular and cardiac involvement and emphasises the importance of early recognition of multisystem disease.

## Introduction

Systemic sclerosis is a multiorgan connective tissue disorder that manifests with significant overlap with other rheumatological diseases. The disease is broadly classified into limited and diffuse cutaneous subtypes, with diffuse cutaneous disease generally associated with a greater burden of internal organ involvement and the severity of the disease can vary depending on the specific antibody subtype. The presence of autoantibodies is reported in more than 95% of patients and can be useful for early diagnosis [1]. Anti-fibrillarin antibodies are a rare subtype found in systemic sclerosis and are commonly associated with renal crisis and cardiac involvement [1]. This subtype has been identified more commonly in African-American populations and is associated with more severe pulmonary hypertension, bowel involvement, and poor prognosis [2].

We present a case of anti-fibrillarin-positive systemic sclerosis presenting with inflammatory myositis and perimyocarditis, highlighting the diagnostic challenges associated with multisystem disease.

## Case presentation

A 45-year-old man with no significant past medical history was admitted with progressive joint pain, swelling, reduced mobility, and overall decline in function. These symptoms developed on a background of a two-year history of evolving dermatological changes, initially characterised by pruritic depigmented lesions over the scalp and back, which gradually extended to involve the chest, upper limbs, groin, and genital region.

Over the preceding six months, he developed progressive proximal muscle weakness, affecting his ability to climb stairs or undertake activity with his arms raised. This was accompanied by reduced grip strength, painful fissuring of the fingertips, and increasing difficulty with activities of daily living. He also reported bilateral periorbital swelling, finger puffiness, morning eye discharge, and widespread joint pain involving the shoulders, hands, and knees. The morning eye discharge was considered incidental and was not felt to be a manifestation of systemic sclerosis; blepharitis, anterior uveitis, and allergy were considered as possible differential diagnoses. In addition, he experienced dyspnoea on exertion, pleuritic chest pain, and unintentional weight loss of approximately five kilograms over one month, associated with anorexia. There were no other constitutional symptoms, including a history of fever or night sweats present suggestive of infection or malignancy. He was a non-smoker and did not consume alcohol. He had relocated to the United Kingdom in 2021, having previously resided in Nigeria. There was no family history of autoimmune or dermatological disease.

On examination, there were widespread hypopigmented and sclerotic skin changes involving the scalp, trunk, and upper limbs, with areas of skin tightening particularly evident over the fingers. The fingers were also puffy, with fingertip tenderness, skin peeling, and areas of fissuring. The characteristic cutaneous findings are demonstrated in Figures 1, 2. Periorbital oedema was present. There was evidence of active synovitis. No peripheral oedema was observed. Cardiovascular and respiratory examinations were unremarkable.

**Figure 1:**
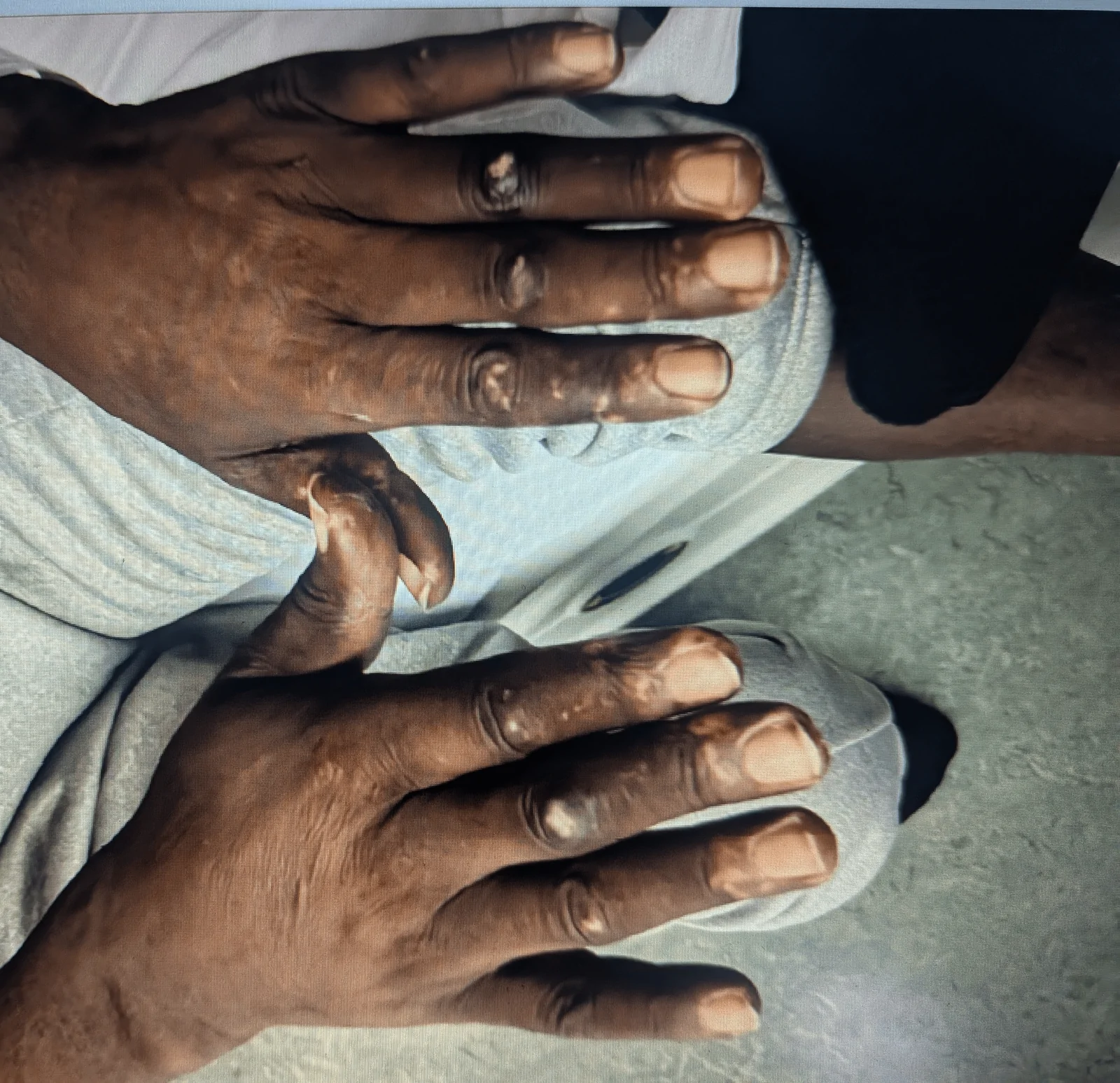
Clinical features of the hands Clinical photograph taken on admission (3-4 months post symptom onset) demonstrating sclerodactyly, digital ulceration and areas of hypopigmentation of the digits bilaterally.

**Figure 2:**
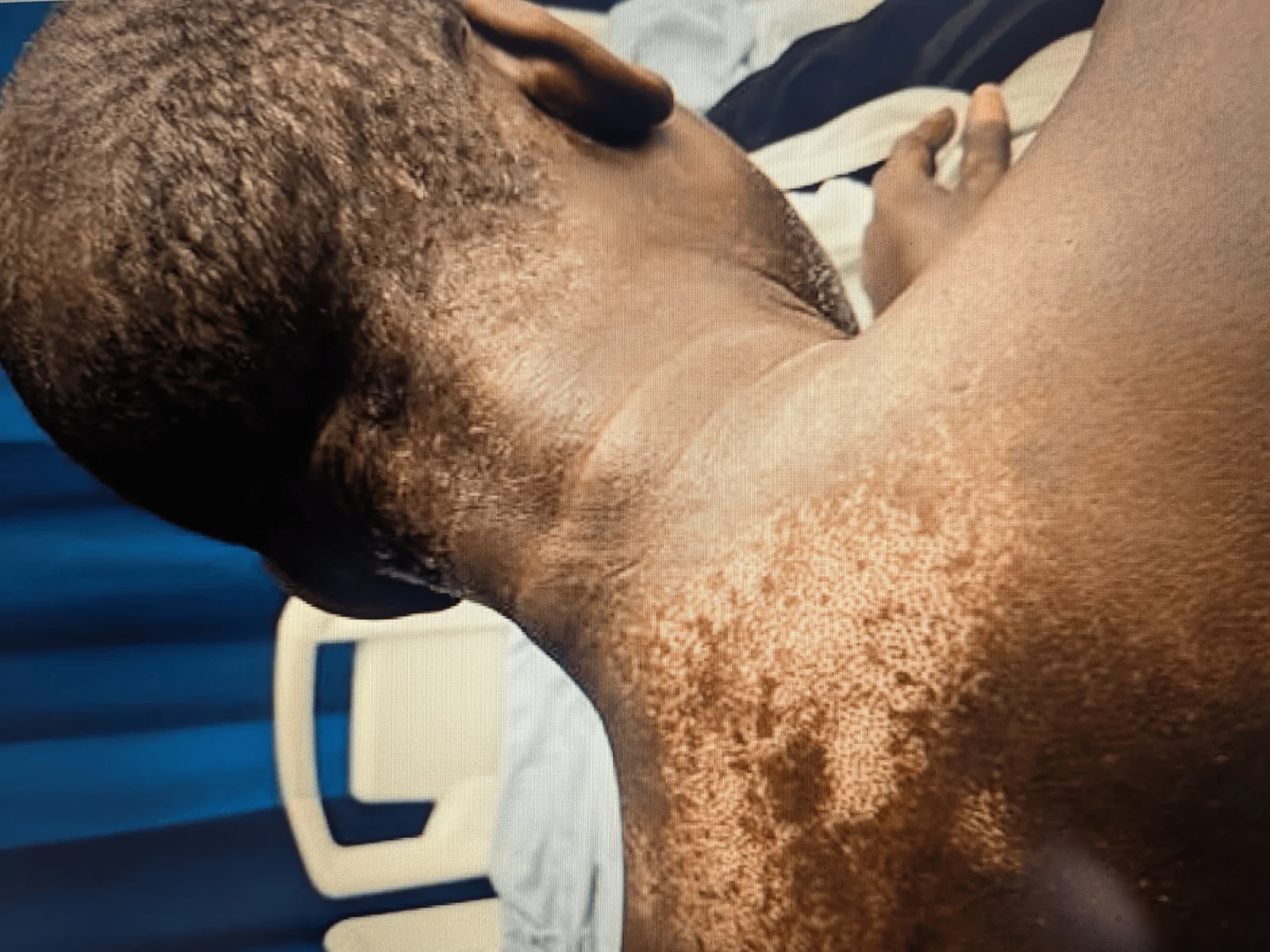
Hypopigmentation on the scalp and upper back Clinical photograph taken on admission (3-4 months post symptom onset) showing widespread hypopigmented skin changes involving the scalp which progressed to the upper part of the back.

Investigations 

A summary of the key laboratory, cardiac, radiological, and functional investigations is provided in the tables below. Initial investigations (Table 1) demonstrated mild skeletal muscle injury and cardiac biomarker elevation. Autoimmune testing was largely negative, apart from anti-fibrillarin antibody positivity (Table 2) on the scleroderma extractable nuclear antigen (ENA) panel. Cardiac magnetic resonance imaging (MRI) demonstrated mild perimyocarditis (Table 3), while thigh MRI showed changes consistent with inflammatory myopathy (Table 4).

**Table 1:** Laboratory investigations CK: creatine kinase; CRP: C-reactive protein; NT-proBNP: N-terminal pro-B-type natriuretic peptide.

Investigation	Result	Reference Range
CK	331 U/L	20 - 200 U/L
CRP	Improved from 5 mg/L to 1.3 mg/L	< 3 mg/L
ESR	Within normal limits	0–20 mm/h
High-sensitivity troponin	Peaked at 121 ng/L, subsequently decreased to 96 ng/L	< 14 ng/L
NT-proBNP	844 ng/L	< 125 ng/L

**Table 2:** Autoimmune laboratory investigations Anti-CCP: Anti-cyclic citrullinated peptide; ANCA: antineutrophil cytoplasmic antibodies; ENA: extractable nuclear antigen; HIV: human immunodeficiency virus.

Investigation	Result
Anti-CCP antibodies	Negative
Rheumatoid factor	Negative
ANCA	Negative
Myositis-specific antibody panel	Negative
Scleroderma ENA panel	Positive anti-fibrillarin antibodies
Complement levels (C3/C4)	Normal
HIV serology	Negative
Hepatitis B serology	Negative
Hepatitis C serology	Negative
Syphilis serology	Negative
Sickle cell screen	Positive

**Table 3:** Cardiac investigations ECG: electrocardiogram, MRI: magnetic resonance imaging.

Investigation	Findings
ECG	Sinus rhythm with first-degree atrioventricular block, anterior T-wave inversion, and left axis deviation
Chest radiograph	Cardiomegaly with cardiothoracic ratio of 57% and rounded cardiac silhouette
Transthoracic echocardiography	Preserved biventricular systolic function
Cardiac MRI	Myocardial oedema with small pericardial effusion; no evidence of myocardial fibrosis or scar formation

**Table 4:** Imaging & functional investigations MRI: magnetic resonance imaging; CT: computed tomography.

Investigation	Result
MRI thighs	Symmetrical muscle oedema involving the iliopsoas, gluteal, and proximal adductor muscle groups
CT pulmonary angiography	No evidence of pulmonary embolism
CT abdomen and pelvis	No evidence of malignancy or lymphadenopathy
Pulmonary function tests	Mild restrictive ventilatory defect with preserved gas transfer

Histopathological examination of a skin biopsy demonstrated dermal fibrosis with attenuation of adnexal structures and minimal chronic inflammatory infiltrate, findings consistent with a scleroderma-spectrum disorder. There was no evidence of malignancy.

Hospital course and management

The initial differential diagnosis included systemic sclerosis, dermatomyositis, and mixed connective tissue disease because of the combination of cutaneous changes, proximal muscle weakness, and inflammatory arthralgia. Dermatomyositis was considered particularly because of the proximal weakness and characteristic hand changes, although the myositis antibody panel was negative and the skin biopsy demonstrated dermal fibrosis and sclerosis. Mixed connective tissue disease was considered but was less likely in the absence of anti-U1 ribonucleoprotein (anti-U1-RNP) antibodies. Systemic lupus erythematosus (SLE) and vasculitic disease were considered but were unsupported by negative double-stranded deoxyribonucleic acid (dsDNA) and antineutrophil cytoplasmic antibodies (ANCA) testing, normal complement levels, and the absence of other characteristic clinical features. Malignancy was investigated with computed tomography (CT) of the chest, abdomen and pelvis, which showed no evidence of malignancy or lymphadenopathy.

During admission, the patient was managed for suspected connective tissue disease with multisystem involvement. In the context of pleuritic chest pain, elevated cardiac biomarkers, and imaging findings, a diagnosis of perimyocarditis was made.

The diagnostic process was complicated by the coexistence of clinical features suggestive of both systemic sclerosis and inflammatory myopathy. The presence of proximal muscle weakness, hand changes, and MRI evidence of muscle inflammation initially raised the possibility of dermatomyositis, while the sclerotic skin changes, sclerodactyly, and digital ulceration suggested systemic sclerosis. This distinction was further complicated by negative myositis-specific serology, which caused delayed recognition. The subsequent identification of anti-fibrillarin antibodies, together with the cutaneous phenotype, supported a diagnosis of systemic sclerosis with inflammatory myopathy overlap.

He was commenced on colchicine and low-dose prednisolone 10 mg daily to address the inflammatory myopathy and associated systemic symptoms, with the potential risk of glucocorticoid-associated scleroderma renal crisis considered. Blood pressure and renal function were monitored during treatment, with a plan to minimise steroid exposure and transition to steroid-sparing therapy post-discharge.

Immunosuppressive therapy was planned by the rheumatology team; however, QuantiFERON-TB (QIAGEN GmbH, Hilden, Germany) testing was positive, indicating likely latent tuberculosis infection. He was therefore commenced on tuberculosis eradication therapy, first with isoniazid 300 mg daily. One month after commencing Isoniazid treatment, mycophenolate mofetil (MMF) was commenced at 1g twice daily, with dose escalation as tolerated to be decided on subsequent follow-up in clinic. 

## Discussion

Systemic sclerosis is a heterogeneous autoimmune disease characterised by immune dysregulation, vasculopathy, and fibroblast activation leading to excessive extracellular matrix deposition [3]. Clinically, anti-fibrillarin systemic sclerosis presents with variable skin fibrosis and classically cardiac and renal involvement, with prognosis largely determined by the extent of systemic disease and antibody subtype. The overlap between systemic sclerosis and inflammatory myopathy is well recognised but diagnostically challenging, as clinical features may be shared and serological markers can be absent [4] such as what was seen in this case.

Cardiac involvement is a recognised cause of morbidity in systemic sclerosis and may present with myocarditis, pericardial disease, or arrhythmia, sometimes preceding overt systemic features [5]. In this case, the patient presented with shortness of breath and pleuritic chest pain, prompting investigation for pulmonary embolism and myocarditis. Elevated troponin, together with electrocardiographic abnormalities and subsequent cardiac MRI findings of myocardial oedema with a small pericardial effusion, demonstrated early myocardial involvement. This presentation is consistent with the recognised phenotype of anti-fibrillarin-positive systemic sclerosis, in which cardiac involvement is more common and may occur early in the disease course, myocardial fibrosis or scar formation would have been indicative of further disease progression.

Anti-fibrillarin antibodies are a rare serological marker, present in approximately 3.5% of patients with systemic sclerosis, and are associated with diffuse disease, greater internal organ involvement, and shorter survival independently of demographic factors and disease type [1, 6, 7]. The prevalence of Anti-fibrillarin antibodies appears to vary according to ethnicity. Previous studies have highlighted that patients of native north American descent are more likely to be positive for these antibodies, while patients of African descent have shown a trend towards a higher frequency of Anti-fibrillarin antibodies [7]. These findings suggest that ethnic background may influence prevalence of anti-fibrillarin positive systemic sclerosis; however, variance (differences) in sample size and population characteristics across studies should be considered when interpreting these findings. Their presence can complicate diagnosis due to overlap with other connective tissue diseases and the absence of a single dominant clinical pattern, making early diagnosis challenging, particularly when multiple systems are involved simultaneously, as in this case.

Management in such overlap disease is largely guided by organ involvement, with Immunosuppressive therapy often used as a first-line steroid-sparing agent in patients with inflammatory musculoskeletal and cutaneous disease. Alternative immunosuppressive options, including cyclophosphamide and rituximab, may also be considered depending on the pattern and severity of multi-organ involvement [8]. MMF was chosen in this case as a suitable steroid-sparing option for the management of both inflammatory myopathy and systemic features alongside low-dose corticosteroids. The presence of latent tuberculosis delayed the initiation of MMF until the patient could be well established on anti-TB therapy.

## Conclusions

This case demonstrates the simultaneous presentation of cutaneous sclerosis, inflammatory myopathy, and perimyocarditis in anti-fibrillarin-positive systemic sclerosis. The combination of progressive sclerotic skin changes, proximal muscle weakness, and cardiac involvement should prompt consideration of systemic sclerosis overlap disease, even when initial myositis-specific serology is negative. Early multidisciplinary assessment is important to recognise and evaluate multisystem involvement and guide appropriate immunomodulatory treatment.
